# Introduction: Venezuela, Dispersed: Interdisciplinary Perspectives on Venezuelan Migration

**DOI:** 10.1111/blar.13618

**Published:** 2025-01-10

**Authors:** Rebecca Irons, Katie Brown

**Affiliations:** University College London, London, UK; University of Exeter, Exeter, UK

With over 7.7 million people displaced from Venezuela as of June 2024, over a million of whom are seeking asylum (UNHCR 2024), this is the second largest migratory crisis in the contemporary world, and the most significant in the Americas. Of this number, 6.5 million Venezuelans are now residing in Latin America and the Caribbean (R4V 2024), making this a regional emergency of major, if internationally overlooked, concern.

While Venezuelans have been emigrating over the last two decades since Hugo Chávez came to power in 1999 and began the ‘Bolivarian Revolution’, the 1–2 million who had left by 2014 were predominantly from the educated middle and upper classes, leaving due to political opposition to the regime, a lack of opportunities for skilled work and high levels of crime and violence (Paéz 2015). The unprecedented scale of displacement since 2015 reveals a shifting dynamic, as a much broader section of the population flees a multidimensional crisis, including hyperinflation, shortages of food and medicine and increasing authoritarianism under Nicolás Maduro, who succeeded Chávez after the latter’s death in 2013. The articles gathered in this special section evidence both the push factors from Venezuela and the conditions that the displaced experience in recipient countries. Access to healthcare, for example, is a recurrent concern raised across these articles, both in Venezuela and abroad. Colombia hosts the highest number of Venezuelans at 2.8 million as of January 2024 (IOM 2024). As such, it is appropriate that two of the articles focus on Venezuelan migration to and within the Colombian cities of Bogotá (Irons), Bucaramanga and Medellín (Wilde et al.). The third article by Lines et al. discusses migration to Brazil, which hosts around 20 per cent of the Venezuelan diaspora (ACAPS 2023).

The relatively recent surge in displacement has presented a fast-changing landscape that necessitates an interdisciplinary lens to better understand and address the ongoing emergency. To collaboratively address the intersecting issues arising, in May 2022 we brought together 22 international scholars, in-person and online, at ‘Venezuela, Dispersed: Interdisciplinary Perspectives on Venezuelan Migration and the Diaspora’, a conference held at the University of Exeter, UK, with funding from the Institute of Modern Languages Research. The far-reaching implications of Venezuelan displacement are evident from the breadth of subjects explored at the conference, including the cultural rights, literary production and media representations of Venezuelan migrants; political mobilisation, asylum seeking and everyday survival; access to healthcare (particularly sexual and reproductive care); creativity, work and intersectionality; and oral testimony and autobiography. The three articles forming this Special Section all began as presentations at this conference. Together, they approach displacement from Venezuela from multiple perspectives, across disciplines including Anthropology, Global Health, Politics, Gender Studies and Human Geography.

Amongst the tides of migrants leaving Venezuela, one particular group of note are the *caminantes* (walkers). This term refers to the population who have left Venezuela on foot, with numbers peaking during the COVID-19 pandemic. To illustrate, between July and August 2020 alone, 100,000 migrants were documented crossing the Venezuela-Colombia border (Pavón Hernández and Ramírez Moncaleano 2022: 41). Though their presence has reduced since then, the existence of the *caminantes* highlights the highly transient nature of the diaspora during the pandemic peak. In response to the unique constraints imposed on undertaking research with migrants in a pandemic context, the featured articles present and develop innovative methods of participatory research with the Venezuelan diaspora, centring the voices of individuals.

Wilde et al. (2024) combined semi-structured interviews with long-term ethnographic fieldwork, maintained digitally when travel was not possible. Based on analysis of Venezuelans effectively mobilising scant resources despite an insufficient response from the Colombian authorities, the authors introduce the concept of ‘contingent hope’, which they define as ‘an orientation towards the future premised on the desired improvement of specific circumstances’. Their article highlights the strength of cross-border kinship ties as a foundation to this hope, while warning that hope will not last indefinitely without concrete improvements in areas such as access to work and regularisation. Lines et al. (2025) approached the challenges of working with displaced Venezuelan women in Brazil through employing the photovoice methodology. Through giving female participants a camera and asking them to depict life through their own ‘lens’, the authors give the migrants themselves control over what story they consider the most important to tell. The findings reveal the broader, social understandings of reproduction and the resultant needs of women beyond the medicalised focus of health researchers and NGOs. Taking a different creative approach, Irons (2025) developed a unique participatory methodology using postcards as a way to communicate with, and document the experiences of, displaced Venezuelans living in Bogotá, Colombia. By asking participants to record the handwritten snapshots of life that they considered to be the most urgent, Irons reframes the colonial medium of the postcard as a decolonial methodology that can support marginalised groups to lead with the information they perceive to be key – rather than the researcher imposing a predetermined framework on the data. Though these works correspond to Venezuela’s emergency, the methods used could inspire other researchers seeking to develop creative, participatory methods while working with hard-to-reach communities in difficult times.

Another common thread across the articles is attention to the gendered nature of migration and to how parenthood shapes both decisions to leave and experiences of displacement. Lines et al. focus on their participants’ roles as carers, which can be both a source of strength and a cause of depletion and anxiety. Wilde et al., meanwhile, demonstrate how children can be an ‘emotional investment in the future’ that fosters contingent hope. This idea reappears in Iron’s article, where helping one’s children is motivation for migration. While there are commonalities across the three articles, their attention to the nuances of lived experiences among Venezuelan migrants are a timely reminder of the heterogeneity that can be lost in overarching accounts of ‘the diaspora’.

## Data Availability

Research data are not shared.
